# Procedure duration and early postoperative stroke after endovascular and hybrid aortic arch reconstruction for acute aortic syndrome: a single-center retrospective cohort study

**DOI:** 10.3389/fcvm.2026.1772103

**Published:** 2026-03-20

**Authors:** Haoze Li, Junzhou Pu, Wenhui Wu, Yiqi Jin, Zhang Cheng

**Affiliations:** 1 Interventional Diagnosis and Treatment Center, Mudanjiang Cardiovascular Disease Hospital, Mudanjiang, Heilongjiang Province, China; 2 Interventional Center of Valvular Heart Disease, Beijing Anzhen Hospital, Capital Medical University, Beijing, China; 3 Department of Interventional Vascular, Affiliated Suzhou Hospital of Nanjing Medical University, Suzhou, China

**Keywords:** acute aortic syndrome, aortic arch reconstruction, hybrid surgery, postoperative stroke, procedure duration, thoracic endovascular aortic repair

## Abstract

**Objective:**

Acute aortic syndrome (AAS) with an inadequate proximal landing zone often requires aortic arch reconstruction using endovascular branch techniques or hybrid supra-aortic debranching plus TEVAR. Despite technical advances, early postoperative stroke remains a clinically important complication. Procedure duration may reflect procedural complexity and cumulative cerebral embolic/hypoperfusion burden, yet its association with early stroke after contemporary arch reconstruction strategies is not well characterized.

**Methods:**

We retrospectively analyzed consecutive AAS patients undergoing aortic arch reconstruction with either Zone 2 single-branch stent-assisted TEVAR or hybrid surgery (supra-aortic bypass with subsequent TEVAR) between January 2017 and December 2023. The primary endpoint was ischemic stroke within 30 days after the procedure, confirmed by imaging. We evaluated the relationship between procedure duration and early stroke using multivariable regression models adjusting for key clinical confounders (age, sex, BMI, hypertension, diabetes, coronary artery disease, prior stroke, and renal insufficiency). Discriminative performance for procedure time was explored using ROC analysis.

**Results:**

A total of 235 patients were included (mean age 60.7 ± 10.6 years; 84.7% male). Procedure duration was longer in the hybrid group than in the Zone 2 single-branch TEVAR group (249.0 ± 72.3 vs. 110.4 ± 43.3 min, *P* < 0.001). Early postoperative stroke occurred in 17 patients (7.2%), with a higher incidence in the hybrid group than in the Zone 2 single-branch TEVAR group (11.1% vs. 3.9%, *P* = 0.034). Procedure duration was independently associated with early stroke after adjustment (adjusted HR 1.012 per minute, 95% CI 1.006–1.018; *P* < 0.001). In exploratory analyses, a procedure-time cut-point of 273 min showed good discrimination for early stroke (AUC = 0.81).

**Conclusions:**

In this single-center cohort of AAS patients undergoing aortic arch reconstruction, longer procedure duration was associated with a higher risk of early postoperative stroke, independent of measured comorbidities. Procedure duration may serve as a pragmatic marker of procedural complexity and cerebral risk exposure, supporting quality-improvement efforts aimed at workflow optimization and risk stratification.

## Highlights

- In patients with acute aortic syndrome undergoing aortic arch reconstruction, longer procedure duration was associated with a higher risk of early postoperative ischemic stroke.
- Procedure duration may serve as a pragmatic surrogate marker of procedural complexity and cumulative cerebral risk during contemporary endovascular and hybrid arch interventions.
- Awareness of prolonged procedure duration may aid intraoperative decision-making, postoperative neurological surveillance, and quality-improvement efforts in aortic arch reconstruction.

## Introduction

Acute aortic syndrome (AAS), including acute aortic dissection, intramural hematoma, and penetrating aortic ulcer, remains a life-threatening condition requiring rapid diagnosis and timely intervention (1–5). When arch involvement or an inadequate proximal landing zone precludes standard thoracic endovascular aortic repair (TEVAR), aortic arch reconstruction strategies are often necessary to achieve durable proximal sealing while maintaining supra-aortic perfusion. With the increasing adoption of endovascular and hybrid approaches for arch pathology, preventing perioperative neurological complications has become a central priority.

Postoperative ischemic stroke after arch reconstruction is particularly devastating, increasing early mortality, prolonging hospitalization, and impairing long-term functional outcomes. Contemporary techniques—including fenestration, chimney, branched devices, and hybrid supra-aortic debranching—enable treatment in anatomically challenging settings (6–11), yet stroke rates remain variable across studies, reflecting differences in patient selection, arch atheroma burden, procedural steps, device deployment, and cerebral perfusion management. Importantly, direct comparisons of early stroke risk between commonly used real-world strategies—such as single-branch endovascular reconstruction vs. hybrid debranching plus TEVAR—remain limited (12), especially in AAS populations with urgent timing and heterogeneous anatomy.

Procedure duration is an easily measurable intraoperative metric and may capture several interrelated contributors to cerebral injury risk, including the number and complexity of procedural steps, prolonged catheter/wire manipulation in the arch, repeated device exchanges, and longer exposure to hemodynamic instability or cerebral hypoperfusion. Although it is unlikely to be a purely causal factor on its own, procedure duration may function as a pragmatic surrogate for cumulative procedural “cerebral insult” and overall complexity. Clarifying the association between procedure duration and early stroke could therefore inform risk stratification, benchmarking, and workflow optimization initiatives, particularly for complex aortic arch reconstructions.

Accordingly, we conducted a single-center retrospective cohort study of consecutive AAS patients undergoing aortic arch reconstruction using either Zone 2 single-branch stent-assisted TEVAR or hybrid supra-aortic debranching with subsequent TEVAR. We aimed to compare early postoperative stroke incidence between these approaches in contemporary practice and evaluate whether procedure duration is independently associated with 30-day ischemic stroke after adjustment for key clinical comorbidities. We further performed exploratory analyses to assess the discriminative performance of procedure duration for early stroke risk.

## Methods

### Study design and ethical approval

This was a single-center, retrospective cohort study conducted at Suzhou Municipal Hospital. The study protocol complied with the Declaration of Helsinki (revised in 2013) and was approved by the institutional ethics committee. The requirement for individual informed consent was waived owing to the retrospective nature of the study, while all patient data were anonymized prior to analysis.

### Study population

**Figure 1:**
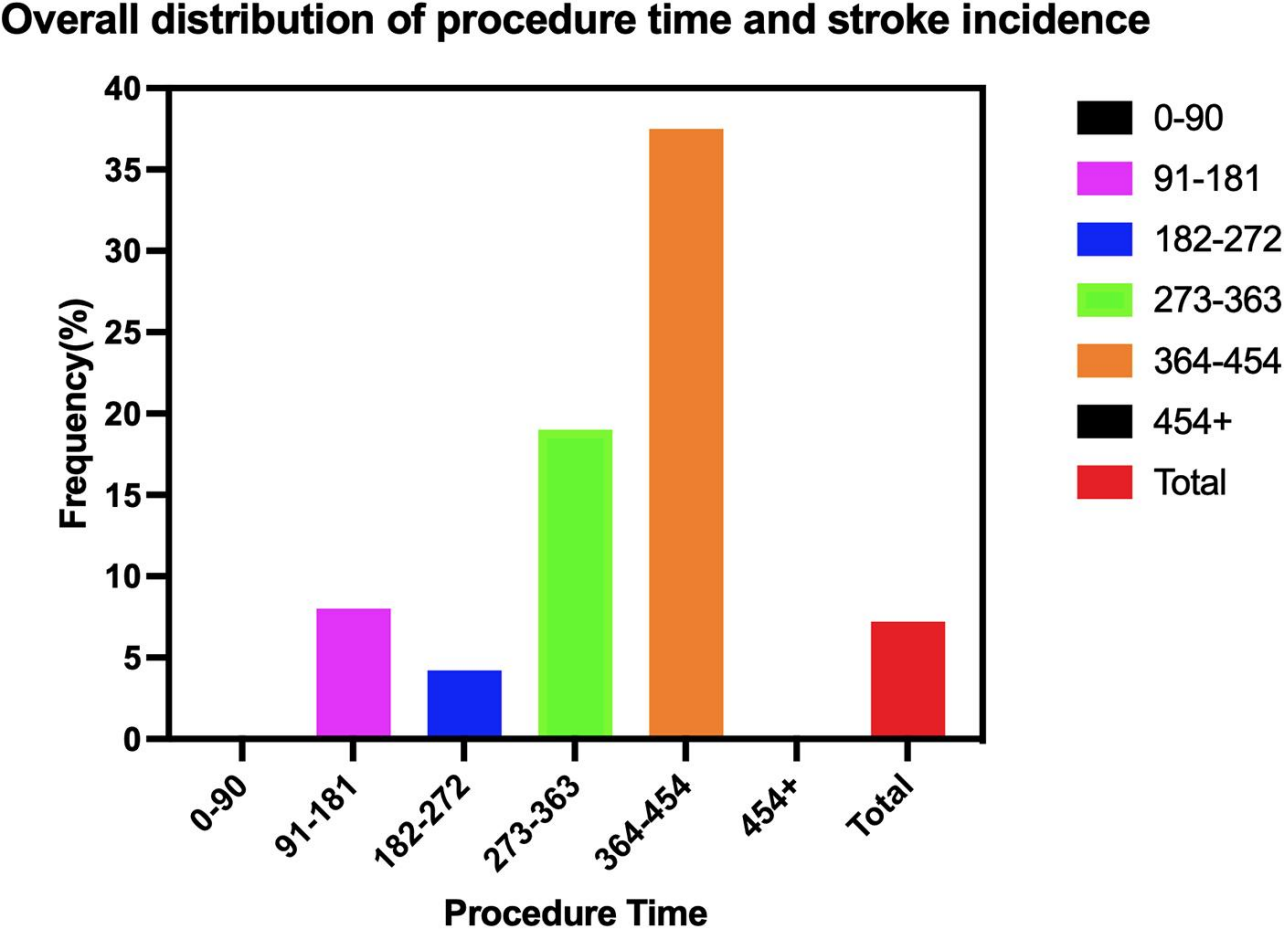
Flowchat.

We retrospectively screened consecutive patients diagnosed with acute aortic syndrome (AAS) who underwent aortic arch reconstruction between January 2017 and December 2023. AAS was defined according to contemporary ACC/AHA and ESC guidelines as acute aortic dissection, intramural hematoma, or penetrating aortic ulcer with symptom onset within 14 days. A patient flow diagram detailing cohort selection and analysis is provided in Figure 1.

Patients were included if they met the following criteria: Presence of AAS with an inadequate proximal landing zone for Zone 2 TEVAR; Underwent aortic arch reconstruction using either single-branch stent-assisted TEVAR or hybrid supra-aortic debranching with subsequent TEVAR; Complete procedural and follow-up data available for analysis.

Patients were excluded if they had hereditary connective tissue disorders (e.g., Marfan syndrome, Loeys–Dietz syndrome), inflammatory aortitis (e.g., Takayasu arteritis), prior aortic arch surgery, or incomplete perioperative records.

## Surgical strategy and procedural techniques

### Surgical strategy selection

The choice of reconstruction strategy was determined by a multidisciplinary aortic team based on anatomical complexity, proximal landing zone location, and overall surgical risk. In general, single-branch stent-assisted TEVAR was favored for patients with Zone 2 landing zones or high cardiopulmonary risk, whereas hybrid surgery was selected for patients with more complex arch anatomy (Zone 0–1 involvement or multiple supra-aortic branches requiring reconstruction).

### Zone 2 single-branch stent-assisted TEVAR

Zone 2 single-branch TEVAR was performed under local anesthesia with conscious sedation in a digital subtraction angiography suite. Vascular access was obtained via bilateral femoral arteries and the left brachial artery. After diagnostic angiography, a femoro-brachial through-and-through wire was established to facilitate accurate deployment of the single-branch stent graft (Castor device). Systemic anticoagulation was achieved with intravenous heparin (6,000 IU). Completion angiography confirmed correct stent positioning, patency of branch vessels, and absence of significant endoleak.

### Hybrid supra-aortic debranching With TEVAR

Hybrid procedures were performed under general anesthesia in a hybrid operating room. Surgical supra-aortic debranching was first completed through bilateral supraclavicular incisions, with bypass grafting between the right subclavian artery and the left subclavian and/or left common carotid artery as indicated. Following successful revascularization, TEVAR was performed to exclude the proximal aortic lesion, intentionally covering the native origins of reconstructed supra-aortic vessels. Embolization of the left subclavian artery was performed when required. Full systemic heparinization was maintained throughout the procedure.

### Definitions and endpoints

The primary endpoint was early postoperative ischemic stroke, defined as a new focal or global neurological deficit lasting more than 24 h and confirmed by computed tomography or magnetic resonance imaging within 30 days after the procedure.

Secondary endpoints included 30-day all-cause mortality, aorta-related adverse events (including retrograde type A aortic dissection, stent graft–induced new entry, aortic rupture), and reintervention during follow-up.

Procedure duration was defined as the time from skin incision or vascular access to final wound closure or sheath removal. It was analyzed as a continuous variable and further explored in categorized and receiver operating characteristic (ROC) analyses.

### Statistical analysis

Continuous variables are presented as mean ± standard deviation and were compared using independent-sample *t*-tests or Mann–Whitney *U*-tests, as appropriate. Categorical variables are expressed as counts and percentages and were compared using chi-square or Fisher's exact tests.

To evaluate the association between procedure duration and early postoperative stroke, univariable analyses were first performed to identify potential risk factors. Variables with *P* < 0.10 in univariable analyses and clinically relevant covariates were entered into multivariable regression models.

Given that stroke events occurred within a short, predefined 30-day postoperative window, Cox proportional hazards models were applied as a time-to-event approach to account for potential variation in event timing within this period. Given the fixed 30-day observation window, multivariable Cox proportional hazards regression models were used to estimate hazard ratios (HRs) and 95% confidence intervals (CIs), with procedure duration treated as a continuous exposure. Sequential models were constructed: Model 1 unadjusted; Model 2 adjusted for age, sex, and body mass index; and Model 3 further adjusted for hypertension, diabetes mellitus, coronary artery disease, prior stroke, and renal insufficiency.

ROC curve analysis was conducted as an exploratory assessment of the discriminative ability of procedure duration for early stroke risk. All statistical tests were two-sided, and *P* < 0.05 was considered statistically significant. Statistical analyses were performed using SPSS version 14.0.

## Results

### Patient characteristics

A total of 235 patients with acute aortic syndrome were included in the final analysis. The mean age was 60.7 ± 10.6 years, and 199 patients (84.7%) were male. Of these, 127 patients underwent Zone 2 single-branch stent-assisted TEVAR, and 108 patients underwent hybrid supra-aortic debranching with subsequent TEVAR.

Baseline demographic and clinical characteristics stratified by reconstruction strategy are summarized in Table 1. Overall, the two groups were comparable in terms of age, sex distribution, body mass index, and most comorbidities. However, patients in the Zone 2 TEVAR group had a higher prevalence of smoking history (*P* = 0.028) and coronary artery disease (*P* = 0.002) compared with those in the hybrid group.

**Table 1:** Baseline characteristics of analysis population with acute aortic syndrome.

Variable	Total (*n* = 235)	Zone 2 TEVAR group (*n* = 127)	Hybrid group (*n* = 108)	*P*-value
Age	60.7 ± 10.6	60.8 ± 10.7	60.6 ± 10.6	0.901
Female	36 (15.3)	24 (18.9)	12 (11.1)	0.099
BMI	26.2 ± 2.8	26.3 ± 2.9	26.0 ± 2.8	0.431
Smoking	127 (54.0)	77 (60.6)	50 (46.3)	0.028
Hypertension				0.387
0	46 (19.6)	20 (15.7)	26 (24.1)	
1	12 (5.1)	6 (4.7)	6 (5.6)	
2	38 (16.2)	23 (18.1)	15 (13.9)	
3	139 (59.1)	78 (61.4)	61 (56.5)	
Diabetes mellitus	34 (14.5)	20 (15.7)	14 (13)	0.469
COPD	11 (4.7)	5 (3.9)	6 (5.6)	0.558
Coronary artery disease	71 (30.2)	49 (38.6)	22 (20.4)	0.002
Stroke	21 (8.9)	12 (9.4)	9 (8.3)	0.765
History of neoplasm	6 (2.6)	4 (3.1)	2 (1.9)	0.690
Previous renal insufﬁciency	20(8.5)	14(11.0)	6(5.6)	0.134

Values are mean ± SD, or *n* (%). TEVAR, thoracic endovascular aortic repair; BMI, body mass index; COPD, chronic obstructive pulmonary disease.

### Procedural and anatomical characteristics

Preoperative imaging characteristics and intraoperative procedural details are presented in Table 2. The mean procedure duration for the entire cohort was 174.1 ± 90.5 min. Procedure duration was significantly longer in the hybrid group than in the Zone 2 single-branch TEVAR group (249.0 ± 72.3 vs. 110.4 ± 43.3 min, *P* < 0.001).

**Table 2:** The imaging anatomical data and procedure information of analysis population with acute aortic syndrome.

Variable	Total (*n* = 235)	Zone 2 TEVAR group (*n* = 127)	Hybrid group(*n* = 108)	*P*-value
Preoperative procedural data				
PLZ diameter (mm)	30.9 ± 3.1	30.9 ± 2.9	30.8 ± 3.3	0.793
Classification of diseases				0.517
Aortic dissection	118 (50.2)	61 (48.0)	57 (52.8)	
Intramural hematoma	34 (14.5)	17 (13.4)	17 (15.7)	
Penetrating ulcer of the aorta	83 (35.3)	49 (38.6)	34 (31.5)	
Aortic arch type, *n* (%)				0.399
I	81 (34.5)	47 (37.0)	34 (31.5)	
II	108 (46)	59 (46.5)	49 (45.4)	
III	46 (19.6)	21 (16.5)	25 (23.1)	
Dominant vertebral artery *n* (%)				0.097
Right dominant	48 (20.4)	24 (18.9)	24 (22.2)	
Equally dominant	61 (26)	27 (21.3)	34 (31.5)	
Left dominant	126 (53.6)	76 (59.8)	50 (46.3)	
Intraoperative procedural data				
Procedure time	174.1 ± 90.5	110.4 ± 43.3	249.0 ± 72.3	<0.001
Covered stent				NA
Relay	26 (11.1)	0 (0)	26 (24.1)	
Medtronic	20 (8.5)	0 (0)	20 (18.5)	
Gore	52 (22.1)	0 (0)	52 (48.1)	
Castor	127 (54.0)	127 (100)	0 (0)	
Others	10 (4.3)	0 (0)	10 (9.3)	
Proximal landing zone (1/2)	46 (19.6)/189 (80.4)	0 (0)/127 (100)	46 (42.6)/62 (57.4)	<0.001
Branch vessel diameter	9.7 ± 1.4	10.8 ± 1.0	8.5 ± 0.8	<0.001

Values are mean ± SD, or *n* (%). TEVAR, thoracic endovascular aortic repair; PLZ, proximal landing zone.

All procedures in the Zone 2 single-branch TEVAR group were performed with a proximal landing zone in Zone 2, whereas the hybrid group more frequently involved Zone 0 or Zone 1 reconstructions (*P* < 0.001). Branch vessel diameter was significantly smaller in the hybrid group, reflecting differences in reconstruction strategy and device selection.

**Figure 2:**
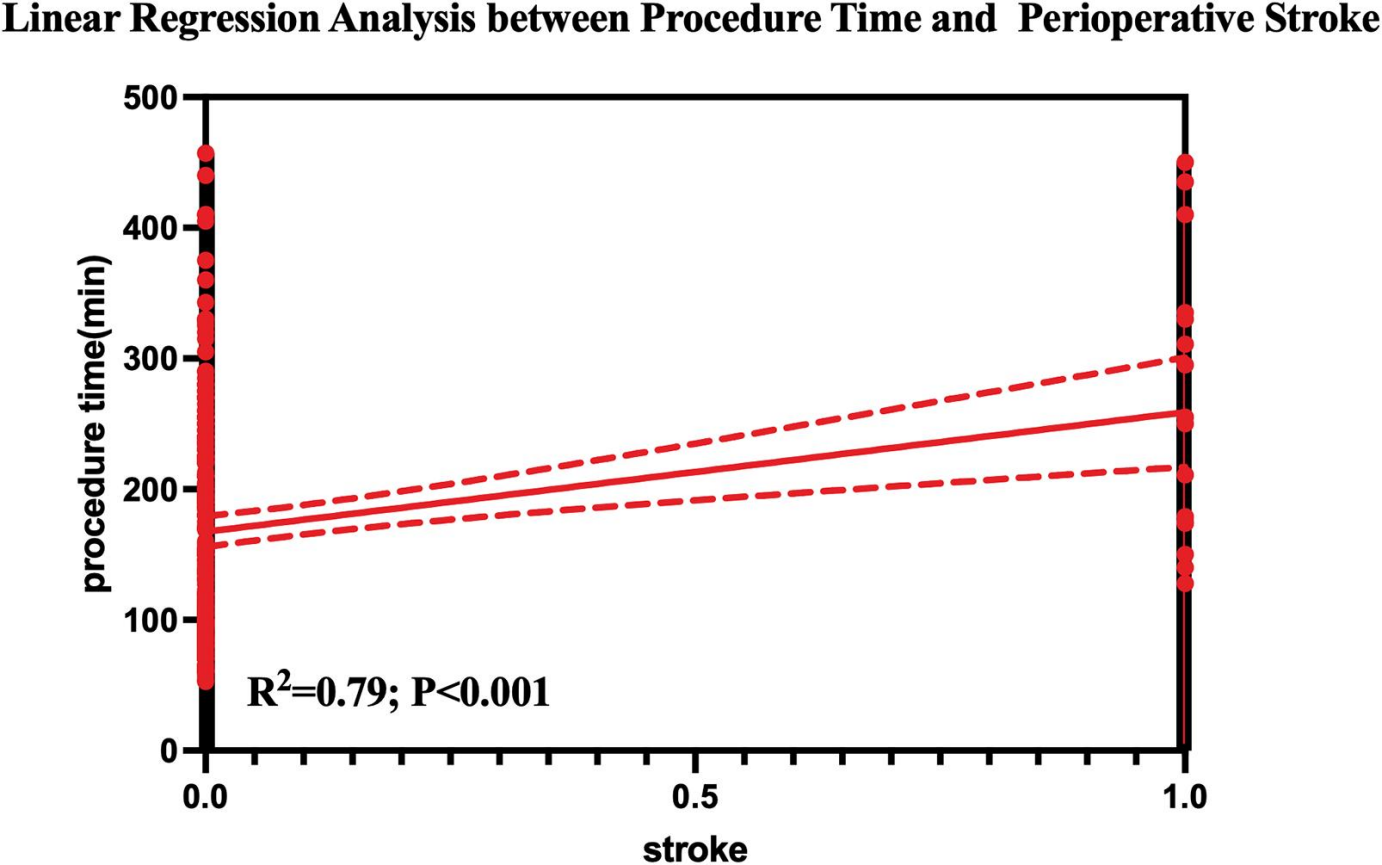
Distribution of procedure time.

The distribution of procedure duration across the entire cohort is illustrated in Figure 2, demonstrating a right-skewed distribution with longer operative times predominantly observed in the hybrid surgery group.

### Early postoperative outcomes

Early postoperative outcomes within 30 days are summarized in Table 3. Early postoperative ischemic stroke occurred in 17 patients (7.2%) overall. The incidence of stroke was significantly higher in the hybrid group compared with the Zone 2 single-branch TEVAR group (11.1% vs. 3.9%, *P* = 0.034).

**Table 3:** Periprocedural complications.

Variable	Total (*n* = 235)	Zone 2 TEVAR group(*n* = 127)	Hybrid group (*n* = 108)	*P*-value
New-onset stroke	17 (7.2)	5 (3.9)	12 (11.1)	0.034
Perioperative mortality	14 (6)	4 (3.1)	10 (9.3)	0.057
Re-intervention	31 (13.2)	12 (9.4)	19 (17.6)	0.066
Bird-beak phenomenon	11 (4.7)	5 (3.9)	6 (5.6)	0.558
Ia endoleak	7 (3.0)	3 (2.4)	4(3.7)	0.706

Values are *n* (%). TEVAR, thoracic endovascular aortic repair.

There were no statistically significant differences between the two groups in terms of 30-day all-cause mortality, reintervention rates, bird-beak phenomenon, or type Ia endoleak.

### Association between procedure duration and early stroke

In univariable analyses, longer procedure duration was significantly associated with an increased risk of early postoperative ischemic stroke. This association remained robust after multivariable adjustment.

Results of Cox proportional hazards regression analyses are presented in Table 4. In the fully adjusted model, which accounted for age, sex, body mass index, hypertension, diabetes mellitus, coronary artery disease, prior stroke, and renal insufficiency, procedure duration remained independently associated with early postoperative stroke (adjusted HR 1.012 per minute, 95% CI 1.006–1.018; *P* < 0.001). In a sensitivity analysis (Model 4), procedure duration remained significantly associated with early stroke even after adjusting for the proximal landing zone (Zone 1 vs. Zone 2), suggesting that operative time reflects cumulative procedural complexity beyond anatomical classification alone.

**Table 4:** Cox proportional hazard ratio (HR) with 95% confidence interval (CI) for stroke of thoracic endovascular aortic repair (TEVAR) patients.

Variable	Model 1		Model 2		Model 3		Model 4	
	HR (95% CI)	*P* Value	HR(95% CI)	*P* Value	HR(95% CI)	*P* Value	HR(95% CI)	*P* Value
Procedure time	1.009 (1.004–1.014)	<0.001	1.010 (1.004–1.015)	<0.001	1.012 (1.006–1.018)	<0.001	1.011 (1.005–1.017)	<0.001

Results were calculated with the use of the measure of procedure time variability as a time-dependent covariates. Continuous body-weight variability is per 1-SD change in procedure time variability. Model 1 was unadjusted. Model 2 was adjusted for age, gender and BMI. Model 3 was adjusted for age, gender, BMI, hypertension, diabetes mellitus, coronary artery disease, stroke and previous renal insufﬁciency. Model 4 was adjusted for age, gender, BMI, hypertension, diabetes mellitus, coronary artery disease, stroke, previous renal insufﬁciency and zone(1/2). BMI, body mass index; CI, indicates confidence interval; LCCA, left common carotid artery; LSA, left subclavian artery; HR, hazard ratio.

**Figure 3:**
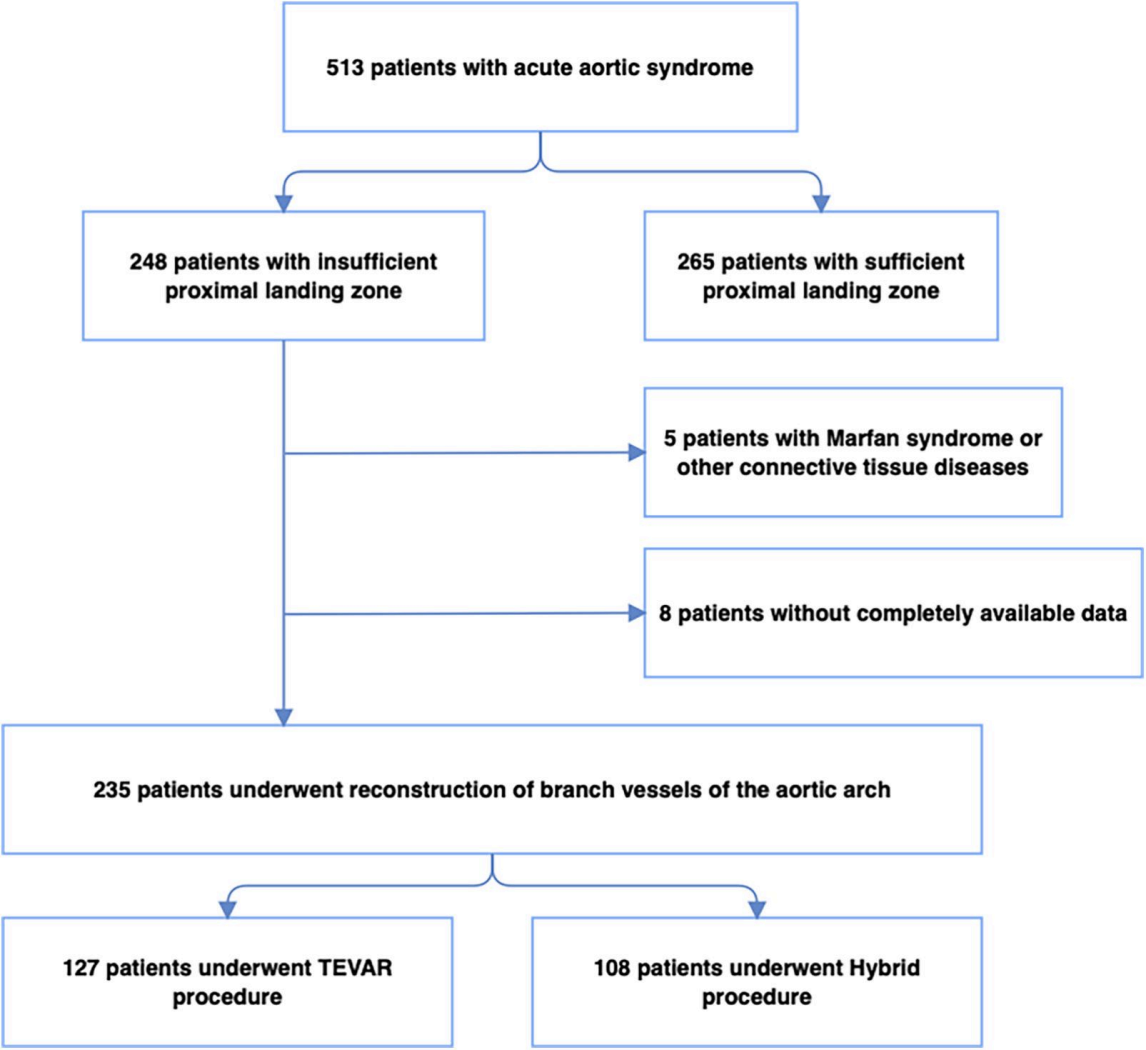
A linear regression between procedure time and stroke.

A linear regression analysis demonstrated a strong positive correlation between procedure duration and stroke incidence (Figure 3). Receiver operating characteristic analysis showed good discriminative performance of procedure duration for early stroke risk (AUC = 0.81). An exploratory time point of approximately 273 min, derived from ROC analysis, was associated with a higher observed incidence of early stroke.

### Stratified analyses

Procedure duration was further categorized into predefined intervals, and stroke incidence across categories is presented in Table 5. Stroke rates increased progressively with longer procedure durations, particularly beyond 273 min.

**Table 5:** Overall incidence of perioperative stroke by categories of procedure time and stratified reconstruction method, gender, and coronary heart disease status.

Characteristic	Procedure time						Total
	0–90 min	91–181min	182–272 min	273–363 min	364–454 min	454 min+	
Overall	0 (0/47)	8.0% (7/87)	4.2% (3/71)	19.0% (4/21)	37.5% (3/8)	0 (0/1)	7.2% (17/235)
Zone 2 TEVAR	0 (0/47)	5.9% (4/68)	8.3% (1/12)	0 (0/0)	0 (0/0)	0 (0/0)	3.9% (5/127)
Hybrid	0 (0/0)	15.8% (3/19)	3.4% (2/59)	19.0% (4/21)	37.5% (3/8)	0 (0/1)	11.1% (12/108)
Women	0 (0/10)	14.3% (2/14)	0 (0/9)	50.0% (1/2)	0 (0/1)	0 (0/0)	8.3% (3/36)
Men	0 (0/37)	6.8% (5/73)	4.8% (3/62)	15.8% (3/19)	42.9% (3/7)	0 (0/1)	7.0% (14/199)
CAD	0 (0/10)	10.8% (4/37)	10.0% (2/20)	66.7% (2/3)	0 (0/1)	0 (0/0)	11.3% (8/71)
Non-CAD	0 (0/37)	6.0% (3/50)	2.0% (1/51)	11.1% (2/18)	42.9% (3/7)	0 (0/1)	5.5% (9/164)

Values are mean ± SD, or *n* (%). CAD, coronary heart disease.

**Figure 4:**
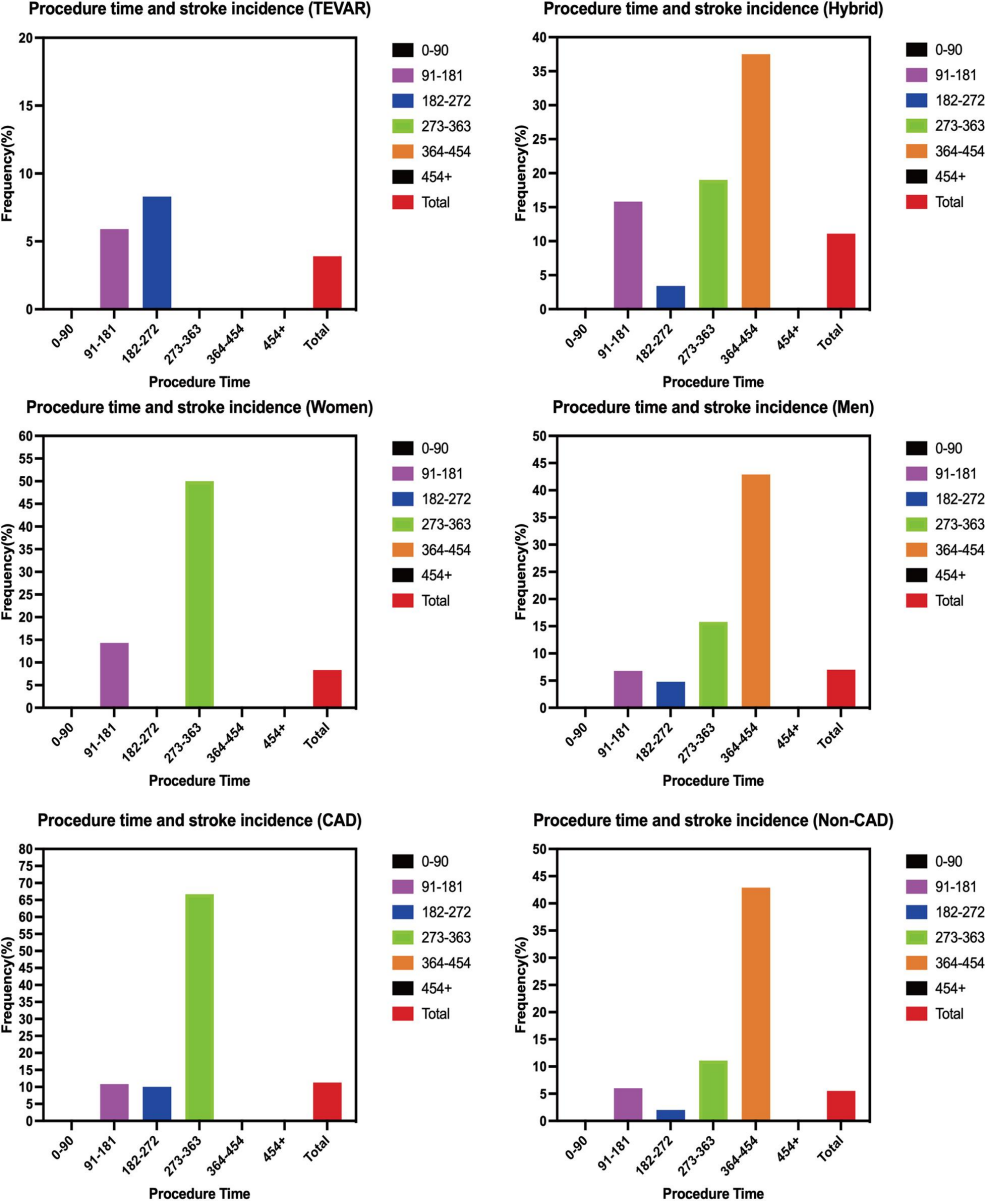
Distribution of procedure time stratified according to operation started, sex and prior coronary heart disease.

When stratified by reconstruction strategy, stroke incidence increased at shorter procedure durations in the Zone 2 single-branch TEVAR group, whereas a more pronounced increase was observed at longer durations in the hybrid group. Additional stratified analyses by sex and coronary artery disease status are illustrated in Figure 4, demonstrating higher stroke susceptibility in female patients and those with pre-existing coronary artery disease during prolonged procedures.

## Discussion

In this single-center retrospective cohort study, we observed a significant association between longer procedure duration and early postoperative ischemic stroke in patients undergoing aortic arch reconstruction for acute aortic syndrome. This association persisted after adjustment for major demographic and clinical confounders, suggesting that procedure duration captures clinically relevant aspects of perioperative neurological risk in contemporary arch reconstruction practice.

Procedure duration itself is unlikely to represent a direct causal factor for stroke. Rather, it likely functions as a composite surrogate marker reflecting procedural complexity, cumulative manipulation within the aortic arch, prolonged exposure to embolic debris, and extended periods of hemodynamic or cerebral perfusion instability (13–15). In hybrid procedures, additional surgical steps, longer general anesthesia time, and the need for supra-aortic bypass reconstruction further amplify these factors, potentially explaining the higher observed stroke incidence in this group.

The findings of this study are consistent with prior reports demonstrating wide variability in stroke rates following endovascular or hybrid aortic arch repair (15–17). While previous investigations have focused primarily on device design (18, 19), arch anatomy, or cerebral protection strategies (20), our results emphasize the importance of procedural efficiency as an integrative measure of technical burden and cerebral risk exposure. Importantly, procedure duration is readily measurable and may therefore serve as a pragmatic quality metric for benchmarking complex arch interventions.

Exploratory analyses suggested that female patients (21–24) and those with pre-existing coronary artery disease may be particularly vulnerable to prolonged procedures. These observations may reflect differences in vascular anatomy, atherosclerotic burden, or physiological reserve, although the limited number of events precludes definitive conclusions. Nonetheless, these findings highlight the potential value of individualized procedural planning and heightened vigilance in high-risk subgroups.

Our study noted a significantly higher prevalence of coronary artery disease (CAD) in the single-branch TEVAR group compared to the hybrid group. This distribution reflects a deliberate risk-stratification strategy by the multidisciplinary aortic team. Patients with severe CAD were preferentially assigned to the totally endovascular approach (single-branch TEVAR) to avoid the physiological stress of open surgical debranching and aortic clamping associated with hybrid procedures. Importantly, although these patients presented with higher cardiovascular risk, this did not necessitate a compromise on anatomical suitability. As indicated by our results, adequate proximal landing zone lengths were achieved in the TEVAR group, ensuring that the ‘less invasive’ choice was not at the expense of sealing stability.

Several limitations merit consideration. The retrospective design and single-center setting limit causal inference and generalizability. Residual confounding related to anatomical complexity, arch atheroma burden, cerebral protection strategies, and intraoperative perfusion parameters could not be fully accounted for. Single-branch TEVAR was favored for high-risk patients with suitable Zone 2 anatomy, while hybrid surgery was reserved for complex arch pathologies. This non-randomized allocation may influence the comparative outcomes. Additionally, exploratory time point derived from ROC analysis should be regarded as hypothesis-generating rather than definitive, given the risk of overfitting and the absence of external validation.

Despite these limitations, this study provides clinically relevant insight into the relationship between procedural characteristics and neurological outcomes in a challenging patient population. Future prospective, multicenter studies incorporating detailed anatomical, hemodynamic, and cerebral monitoring data are warranted to further elucidate mechanisms of stroke and to develop targeted strategies for risk reduction.

## Conclusions

In patients with acute aortic syndrome undergoing aortic arch reconstruction, longer procedure duration was independently associated with an increased risk of early postoperative ischemic stroke. Procedure duration may function as a surrogate marker of procedural complexity and cumulative cerebral risk. These findings support ongoing efforts to optimize procedural strategies and perioperative management to improve neurological outcomes in complex aortic arch interventions.

## Data Availability

The raw data supporting the conclusions of this article will be made available by the authors, without undue reservation.
